# Melatonin Prevents Brain Damage and Neurocognitive Impairment Induced by *Plasmodium Berghei* ANKA Infection in Murine Model of Cerebral Malaria

**DOI:** 10.3389/fcimb.2020.541624

**Published:** 2020-09-30

**Authors:** Brenda Jaqueline de Azevedo Ataide, Nayara Kauffmann, Nívia de Souza Franco Mendes, Marjorie Lujan Marques Torres, Larissa Medeiros dos Anjos, Adelaide da Conceição Fonseca Passos, Suellen Alessandra Soares de Moraes, Evander de Jesus Oliveira Batista, Anderson Manoel Herculano, Karen Renata Herculano Matos Oliveira

**Affiliations:** ^1^Laboratory of Experimental Neuropharmacology, Biological Science Institute, UFPa, Belém, Brazil; ^2^Laboratory of Protozoology, Topical Medicine Nucleus, UFPa, Belém, Brazil

**Keywords:** cerebral malaria, melatonin, blood–brain barrier, neurobehavioral impairment, *Plasmodium*

## Abstract

Cerebral malaria is characterized by permanent cognitive impairments in *Plasmodium*-infected children. Antimalarial therapies show little effectiveness to avoid neurological deficits and brain tissue alterations elicited by severe malaria. Melatonin is a well-recognized endogenous hormone involved in the control of brain functions and maintenance of blood–brain barrier integrity. The current study has evaluated the effect of melatonin on the histological alterations, blood–brain barrier leakage, and neurocognitive impairments in mice developing cerebral malaria. Swiss mice infected with *Plasmodium berghei* ANKA strain was used as cerebral malaria model. Melatonin treatment (5 and 10 mg/kg) was performed for four consecutive days after the infection, and data have shown an increased survival rate in infected mice treated with melatonin. It was also observed that melatonin treatment blocked brain edema and prevented the breakdown of blood–brain barrier induced by the *Plasmodium* infection. Furthermore, hematoxylin and eosin staining revealed that melatonin mitigates the histological alterations in *Plasmodium*-infected animals. Melatonin was also able to prevent motor and cognitive impairments in infected mice. Taken together, these results show for the first time that melatonin treatment prevents histological brain damages and neurocognitive alterations induced by cerebral malaria.

## Introduction

Malaria is a potentially life-threatening disease affecting an estimated 207 million people each year (WHO, 2017). Majority of the fatal cases were due to cerebral malaria (CM), which is the most severe neurological complication of *Plasmodium falciparum* infection that affects mainly children under 5 years of age (WHO, 2000; Idro et al., 2005). Major clinical symptoms of CM include dyspnea, fever, sudden bleeding, disorientation, convulsions, coma, and death. CM survivors could exhibit long-term neurocognitive impairments such as cortical blindness, hearing loss, ataxia, and memory and attention disorders, which is partly due to the fact that antimalarial drugs do not prevent the damages in the central nervous system (CNS) parenchyma (John et al., 2008; Rénia et al., 2012). The mechanism of CM-induced brain injuries is still not fully understood. However, previous studies reveal that increased cytoadherence of parasitized red blood cells (pRBC) leads to brain microvascular obstruction, hypoxia state, and consequent damage of the brain structures. Data from literature also describe the role of proinflammatory cytokines, microglial activation, and oxidative stress on the pathogenicity of CM (Hunt and Grau, 2003).

Most of these neuropathological mechanisms associated with CM were clarified utilizing animal models that simulate the clinical signals described in humans developing CM. Experimental cerebral malaria (ECM) can be developed by using the rodent malaria model with the infection of susceptible mouse strains as C57BL/6 and Swiss albino mice with *Plasmodium berghei* ANKA parasites (Combes et al., 2005; Martins et al., 2009a,b). The mouse model exhibit several of the neurological features of human cerebral malaria (HCM) such as brain neuroinflammation (Hunt and Grau, 2003), reduced cerebral blood flow, blood–brain barrier (BBB) disruption, microhemorrhages (Hunt et al., 2006), brain swelling, and neurological impairment (Desruisseaux et al., 2008; Dai et al., 2010). In this context, the use of endogenous compounds with recognized ability to protect CNS parenchyma could exert significant protection against the brain damage and cognitive impairments evoked by CM.

Melatonin (N-acetyl-5-methoxytryptamine) is an endogenous neuro-hormone primarily synthesized and released from the pineal gland of mammals during the dark phase of the light–dark cycle (Rodriguez et al., 2004). In addition to the pineal gland, melatonin could be released at several extrapineal sites at the CNS, including hypothalamus, cerebellum, retina, nucleus gracilis, medulla oblongata, and cerebral cortex, modulating several neurophysiological roles (Jimenez-Jorge et al., 2007; Radogna et al., 2010). Furthermore, previous studies also describe that melatonin could act as a neuroprotective molecule in both acute brain injuries as cerebral ischemia and chronic neurodegenerative conditions such as Alzheimer and Huntington disease (Wang et al., 2011; Rudnitskaya et al., 2015). Moreover, melatonin could effectively reduce brain inflammation by inhibiting nuclear factor kappa B (NF-κB) translocation and matrix metallopeptidase-9 (MMP-9) activation in lipopolysaccharide (LPS)-induced inflammation in both *in vivo* and *in vitro* experimental models (Chang et al., 2012). This indoleamine is also effective in reducing oxidative stress by increasing the activity and expression of several antioxidant enzymes such as superoxide dismutase, catalase, and glutathione peroxidase (Fischer et al., 2013; Zhang and Zhang, 2014). Melatonin has been implicated in cognitive events as learning process and memory formation as well as ameliorating motor coordination after brain injuries (He et al., 2013; Bavithra et al., 2017). In the rat model of hypoxia–ischemia, melatonin treatment efficiently decreases brain damage by the modulation of BBB integrity.

Recently, a report also demonstrated that melatonin treatment could modulate the progression of infectious disease such as amoebiasis, leishmaniasis, and trypanosomiasis (Daryani et al., 2018). During a rat chronic infection with *Trypanosoma cruzi*, melatonin attenuates oxidative stress condition and the inflammatory process by decreasing nitric oxide and lipid peroxidation and raising the release of interleukin-17A (Brazão et al., 2015).

In malaria infection, melatonin displays a controversial role in the growth and development of some *Plasmodium* species. It has been described that endogenous melatonin modulates the parasite life cycle and maintain the synchronicity of *Plasmodium falciparum and Plasmodium chabaudi*, which assure the propagation of the infection in the host (Beraldo et al., 2005; Budu et al., 2007). In spite of that, a previous study showed that melatonin does not synchronize experimental malaria infection caused by *Plasmodium berghei* and *Plasmodium yoelii* (Bagnaresi et al., 2009). Recent studies have demonstrated that melatonin-derived synthetic indoles, which modulate the activation of melatonin receptors, inhibited *in vitro P. falciparum* growth at lower concentrations (Schuck et al., 2014; Luthra et al., 2019), emerging as new alternative therapeutic approach to alter disease progression. Little is known about the effect of melatonin in the host infected with *Plasmodium*, which mainly concerns the cerebral impairment elicited by the disease.

Taken together, all of these findings prompted us to ask if melatonin treatment is able to modulate the progression of the disease by preventing histological alteration and neurocognitive impairments elicited by CM in mice.

## Materials and Methods

### Experimental Animals

Male Swiss albino mice 5–6 weeks old (weighing 20–24 g) were obtained from the Animal Care Facilities of the Institute of Biological Science, Federal University of Para (Belem-Brazil). Mice were maintained in polypropylene cages (five mice per cage) under standard and controlled conditions of temperature (24 ± 1°C) and humidity (55 ± 10%). Filtered water and standard pellet diet were given *ad libitum*.

###  Plasmodium Berghei ANKA Infection

Infection was performed as previously described by Oliveira et al. (2017). *Plasmodium berghei* ANKA (PbA) strains (Laboratory of Experimental Neuropharmacology, UFPa) were kept as frozen stocks in liquid nitrogen vials. Briefly, all experimental mice were intraperitoneally (i.p.) infected with 1 × 10^6^ PbA-pRBCs suspended in 0.1 ml of phosphate-buffered saline (PBS), obtained from cardiac puncture from homolog mice that had been previously injected with frozen PbA stock solution.

In the course of the disease, mice were observed for parameters as weight loss, survival, blood parasitemia, and ECM clinical signs. Infected and treated animals were assessed daily, and the time of death was promptly registered. Behavioral changes, such as ataxia and convulsion state, were used as established humane endpoints to reduce animal suffering.

Animal body weights were regularly measured during the course of the disease. Parasitemia levels (percentage of pRBCs) were monitored daily by microscopic counting from Giemsa-stained (Sigma-Aldrich) thin smears obtained from mice tail-vein blood and determined according to the formula [(number of pRBCs)/(total numbers of RBCs counted)] × 100.

### Melatonin Treatment

To evaluate the potential neuroprotective effect of melatonin in ECM, mice were randomly assigned into four distinct groups: uninfected control group, PbA-infected group and PbA-infected and treated with 5 mg/kg melatonin, and PbA-infected and treated with 10 mg/kg melatonin. Melatonin (Sigma-Aldrich) dissolved in 1% DMSO (Sigma-Aldrich, Brazil) solution and saline (0.9%; pH 7.4) was given by intraperitoneal injection once daily at a dose of 5 and 10 mg/kg for four consecutive days post-infection, starting on the day of infection. Mice of the control group and PbA-infected group received (i.p.) 0.9% saline solution daily.

### Rapid Murine Coma and Behavior Tests

At day 6 post-infection, the behavioral clinical signs and disease severity was monitored using the grading quantitative Rapid Murine Coma and Behavior scale (RMCBS) described by Carroll et al. (2010). This method was developed to assess the early clinical manifestation of murine CM, and the protocol consists of 10 parameters in which hygiene-related behavior gait, body position, exploratory behavior, and balance were assessed and scored from 0 to 20, in which 0 corresponds to the lowest compromised neurological function and 20 corresponds to severe neurological impairment.

Briefly, in 3 min, the animals were subjected to a video recording for 90 s to assess behavioral parameters such as gait, motor performance, body position, touch escape, pinna reflex, toe pinch, aggression, and grooming. In the next 90 s, mice were assessed for limb strength and balance. Each parameter was scored 0–2 points based on the performance of the infected and treated mice, in which 0 corresponds to severe neurological impairment and 2 corresponds to the lowest compromised neurological function. The total score (0–20) of the domains was calculated by the sum of the parameters for each animal on day of analysis.

### Evans Blue Dye Perfusion for Vascular Leakage

BBB disruption was assessed by measuring Evan's blue extravasation as previously described by Kim et al. (2014). Briefly, at day 6 post-infection, mice were intraperitoneally injected with 200 μl of 1% Evan's blue dye (Sigma-Aldrich) prepared in sterile saline solution. After 2 h, anesthetized mice were perfused with saline and sacrificed. Brains were isolated, weighed, and placed in dimethyl formamide for 48 h at 37°C (in the dark) to extract Evan's blue dye from the brain tissue. The concentration of Evan's Blue dye was measured at 620 nm in a plate reader and calculated using a standard dye curve. Data were expressed as micrograms of dye extravasated per gram of brain tissue.

### Edema

Brain water content as a marker of cerebral edema was determined using the wet/dry method previously described by Ding et al. (2015). At 6 days post-infection, mice were deeply anesthetized and brains were collected and immediately weighted (wet weight) and then dried at 80°C for 72 h (dry weight). The percentage of brain water content was then estimated as [(wet weight – dry weight)/wet weight × 100%].

### Hematoxylin and Eosin Staining and Cell Counting

Mice were first anesthetized with ketamine and xylazine, and the brains of each experimental group were transcardially perfused with PBS and 4% paraformaldehyde. Post-fixed brains were carefully dissected and embedded in paraffin wax. Serial coronal sections into 8 μm were performed and stained with hematoxylin and eosin (H&E). The brain cortex was examined in the cortical–medullary area under a light microscope and photographed at 20, 40, and 60 × objective lens. For cell counting, DAPI (4',6-diamidino-2-phenylindole; Sigma) staining was performed as previously described (Chan et al., 2012). In brief, the free-floating sections were washed with PBS and incubated with DAPI solution (1:10,000) for nuclear staining at 37°C for 2 min. To count the cell nucleus, slices were placed in mounting medium (Fluoroshield) and visualized under fluorescence microscopy (**×20 objective lens**); (Nikon_EclipseNi). To assure the counting of cells from cerebral parenchyma, areas of cell infiltrate were excluded from DAPI counting. Data were analyzed using ImageJ software.

### Open Field Behavior Test

The open field is a common test used to measure mice locomotor and exploratory behavior. The test was performed 6 days post-infection according to the protocol described by Desruisseaux et al. (2008). Briefly, mice were individually placed in the center of the testing chamber (83 × 52 cm) with black floor to freely explore the arena for 5 min. Data recorded included the number of crossed quadrants, grooming frequency, and lifting frequency. Each animal performed only one trial test. The apparatus was cleaned with 70% alcohol between trials, and tests were carried out under the same standard conditions. The sessions were monitored by a digital camera attached above the apparatus, and later analyses were conducted using the X-Plot-Rat Software.

### Statistical Analysis

All data were expressed as the mean ± standard deviation (SD). Statistical analyses were performed using one-way ANOVA followed by post-test Tukey–Kramer. Survival data were compared and analyzed using log-rank test of Kaplan–Meier curves. Mice were randomly divided into groups of 10 animals (per group), and all data are representative of at least two independent experiments. Analyses were conducted with the GraphPad Prism Software and significant differences were defined with *p* values below 0.05.

## Results

### Melatonin Treatment Improves the Survival Rate and Disease Severity in Swiss Mice Infected With PbA

**Figure 1:**
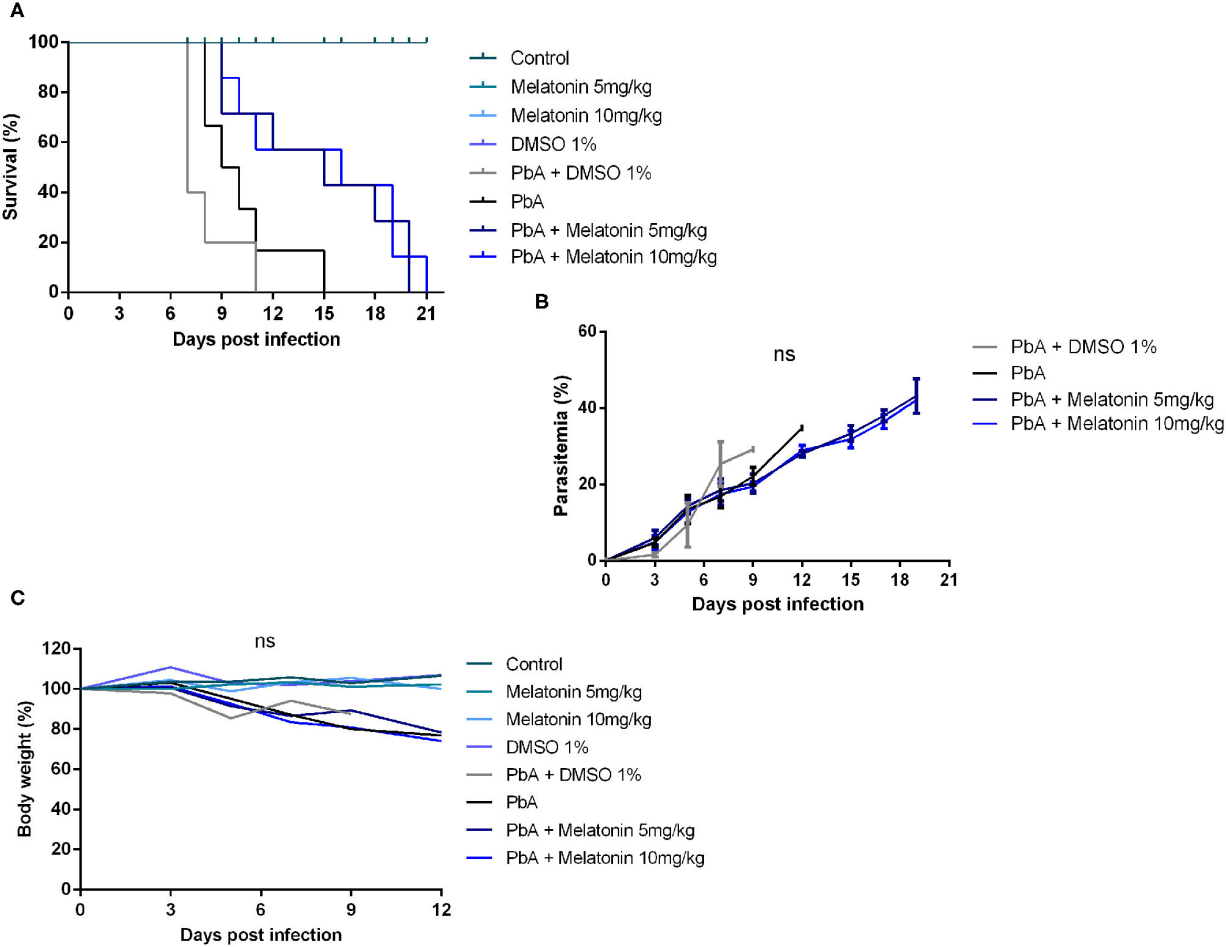
Melatonin prolonged survival of *Plasmodium berghei-*infected mice. *Plasmodium berghei* ANKA (PbA)-infected mice were treated intraperitoneally from day 1 to day 4 post-infection with melatonin at 5 and 10 mg/kg/day. **(A)** Survival curve improved after melatonin treatment compared to saline PbA-infected group (*p* ≤ 0.05, log rank test). **(B)** Parasitemia curve did not differ significantly between groups. Parasitemia levels were measured as the number of parasitized red blood cells (pRBCs) in at least 1,000 RBCs (ns, not significant). **(C)** Body weight variation during the infection (%). The results are representative of three independent infections; *n* = 10 animals/group. Results shown are mean ± SD.

To address the effect of melatonin on the clinical progression of ECM, PbA-infected mice were treated with 5 and 10 mg/kg/day of melatonin for four consecutive days. Consistent with prior observations, PbA-infected mice developed marked neurological symptoms between 6 and 10 days post-infection, characterized by physical inactivity, hemiplegia, and ataxia ending up with convulsions, coma, and death. Eighty percent of PbA-infected mice succumbed to CM on days 8–11 whereas 20% died on day 15 due to severe malaria (Figure 1A). Despite having no anti-parasitic action, melatonin treatment in both doses of 5 and 10 mg/kg significantly prolonged the survival rate of PbA-infected mice. At 11 days post-infection, the melatonin-treated group exhibited a 57% of survival rate with mice showing no CM neurological symptoms when compared to the PbA-infected group that exhibited a 16% survival rate (Figure 1A). To investigate whether the survival improvement was correlated to changes in peripheric parasite rate, parasitemia was monitored, but no significant difference was registered between groups (Figure 1B). Further, melatonin also had no effect on body weight average in uninfected mice or in PbA-infected mice (Figure 1C).

**Figure 2:**
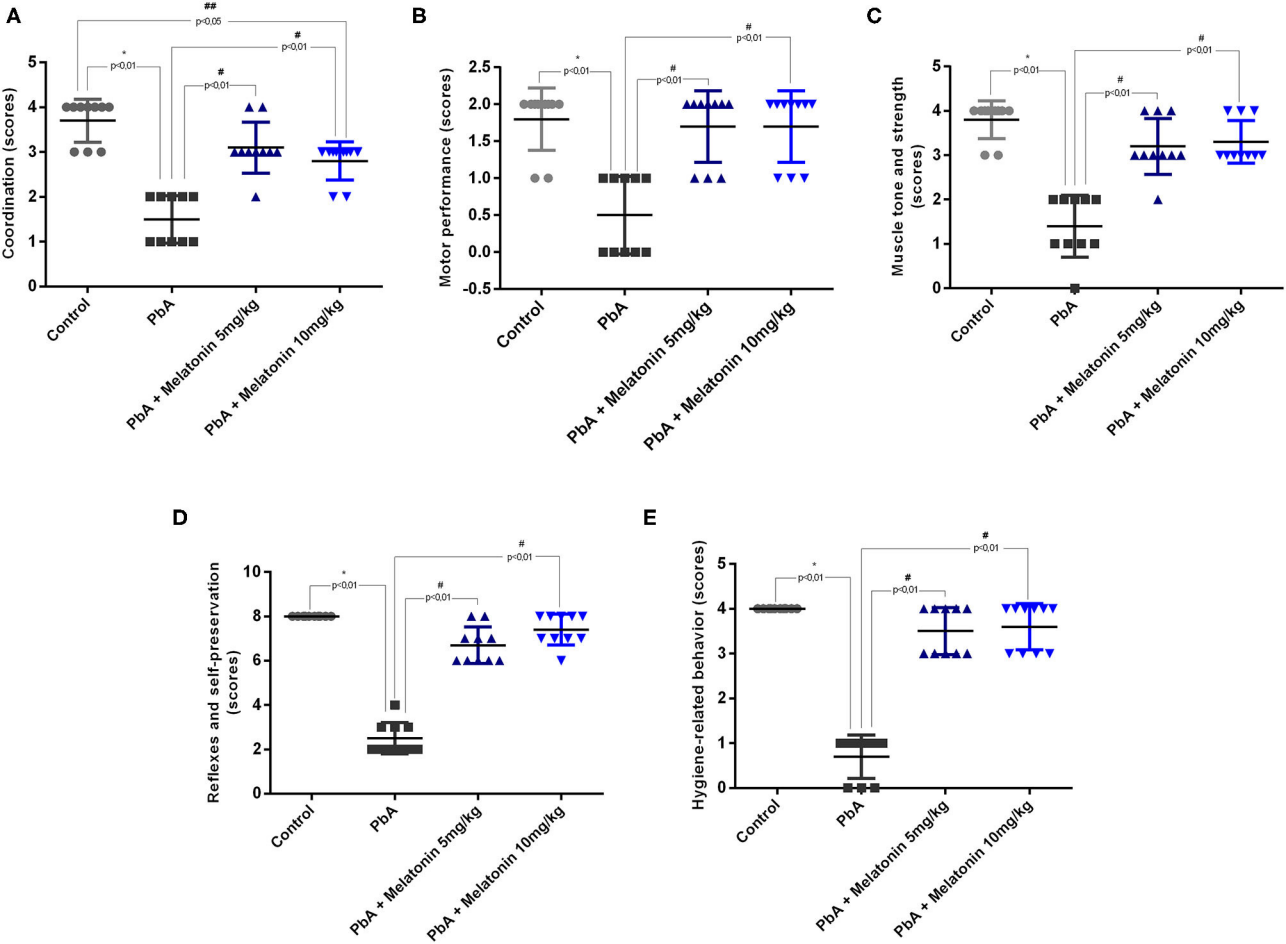
Neurological signs based on RMCBS were significantly reduced in PbA-infected mice at 6 days post-infection. Melatonin treatment improves all the parameters analyzed as coordinating **(A)**, locomotion **(B)**, strength and muscle tone **(C)**, self-preservation **(D)**, and grooming **(E)**; *n* = 10 animals/group. Data are presented as average ± SD. *p < 0.01 vs control; ^#^*p* < 0.01 vs PbA; ^*##*^*p* < 0.05 vs PbA.

In the RMCBS protocol, melatonin treatment also reduced disease severity and the development of neurological signs associated to ECM. It was possible to notice that melatonin treatment at both 5 and 10 mg/kg prevented the loss of all functional domains evaluated in the late stage of the disease (day 6 post-infection) such as coordination, motor performance, muscle tone and strength, reflexes and self-preservation, and hygiene-related behavior (Figure 2).

### Melatonin Prevents BBB Disruption and Cerebral Edema Induced by PbA Infection

**Figure 3:**
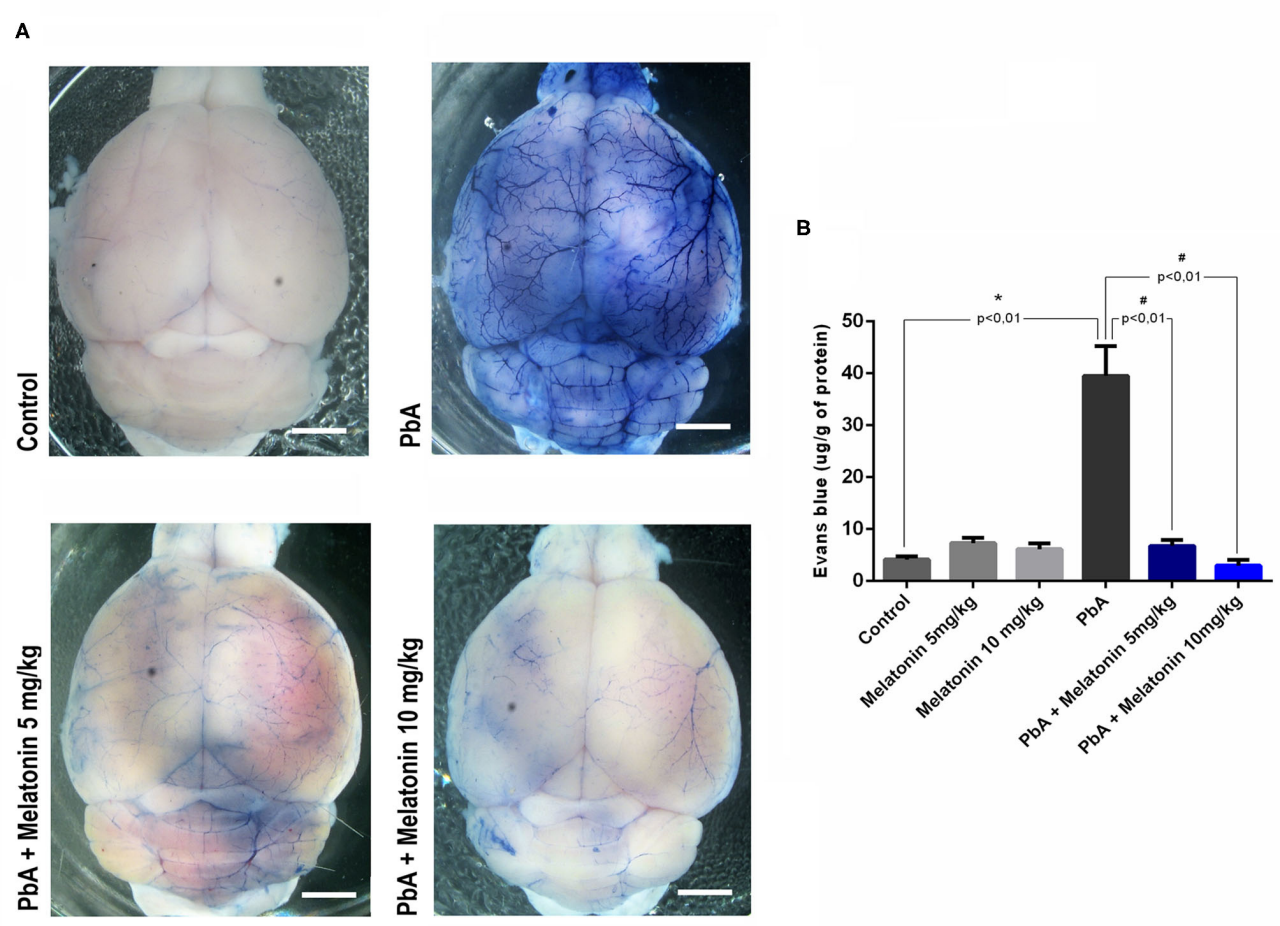
Melatonin maintains blood–brain barrier integrity in ECM. **(A)** Qualitative analysis of whole brain after Evans blue dye administration in uninfected control group, PbA-infected mice, 5 mg/kg melatonin-treated PbA mice, and 10 mg/kg melatonin-treated PbA mice. Scale bars: 0.5 mm. **(B)** Quantification of Evans blue (EB) extravasated into brain at 6 days post-infection. The experiment was repeated three times and subjected to one-way ANOVA and expressed as mean ± SD (*n* = 8, *p < 0.01 vs. control; ^#^*p* < 0.01 vs. PbA).

We next investigated whether brains of melatonin-treated mice showed differences in the stability of the BBB, once its disruption is a crucial event associated with human and experimental CM outcome (de Souza et al., 2009). The effect of melatonin in BBB permeability was evaluated by determining the Evans blue dye concentrations in the mice brain tissue. Figure 3A shows representative images of whole brains in the distinct groups, revealing that melatonin-treated PbA-infected mice had a more discrete staining when compared to the PbA-infected group.

The quantification of formamide extraction revealed that the brains of CM mice had a marked increased in BBB permeability at 6 days post-infection (an average increase of 40% in Evans blue extravasation as compared to uninfected control group—from 8 μg/g in uninfected control to 40 μg/g in PbA-infected mice) (Figure 3B). In contrast, melatonin-treated PbA-infected mice in both doses of 5 and 10 mg/kg exhibited a dramatically reduced amount of Evans blue extravasated into the brain parenchyma when compared to PbA-infected mice (Figure 3B).

**Figure 4:**
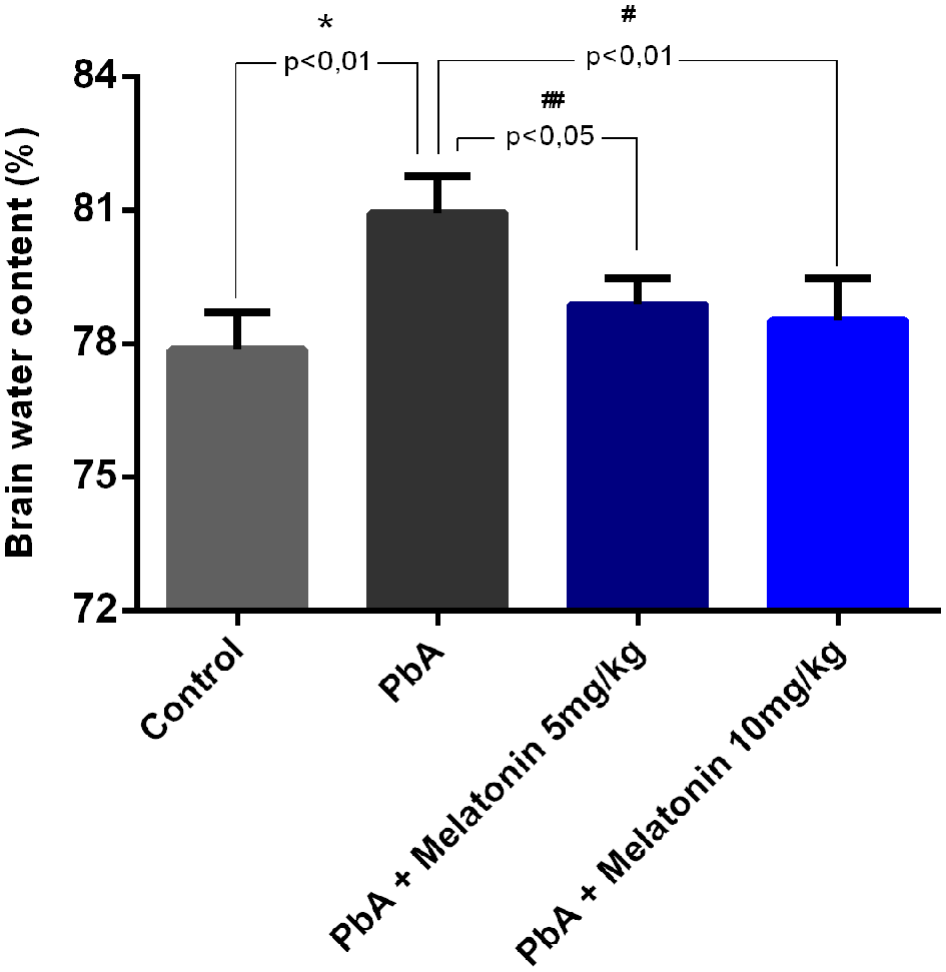
Melatonin therapy alleviated cerebral edema in ECM. Brain water content was measured at 6 days post-infection in different groups (*n* = 8, *p < 0.01 vs. control; ^#^*p* < 0.01 vs. PbA; ^##^*p* < 0.05 vs. PbA).

As shown in Figure 4, brain water content was also evaluated at day 6 post-infection as an indicator of brain edema. It was demonstrated that PbA infection significantly increased brain water content when compared to the uninfected mice group. However, melatonin-treated PbA-infected mice had significantly less brain water content than the PbA-infected group. This result indicates that melatonin ameliorates the brain edema induced by PbA infection.

### Melatonin Decreases Histological Alterations in PbA-Infected Animals

**Figure 5:**
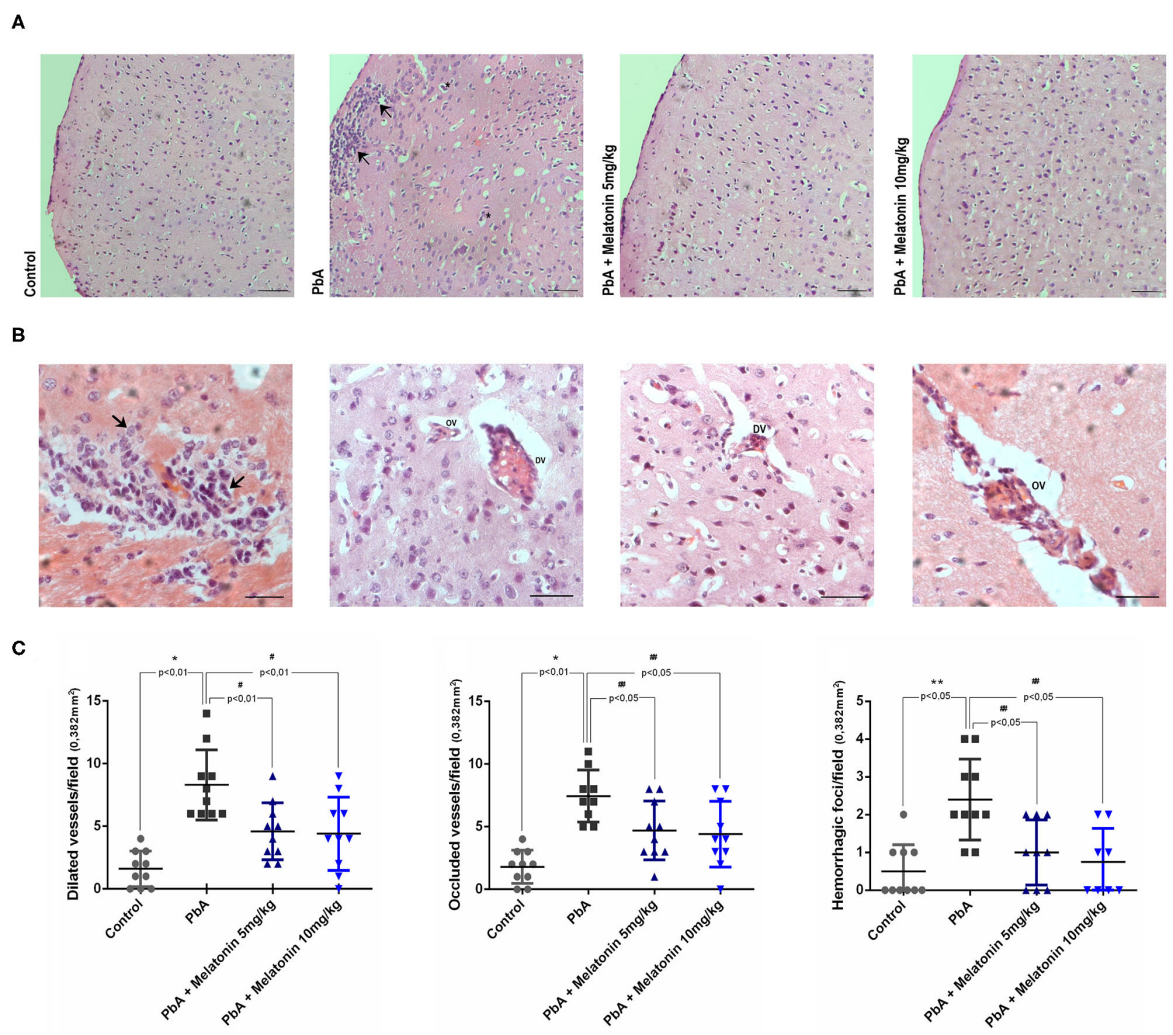
Melatonin treatment decreases histological alteration into the brain tissue of PbA-infected mice. Representative light microphotographs from mice cerebral cortex at day 6 post-infection stained with H&E. **(A)** Brain cortex from uninfected mice (control) with normal histological appearance; PbA-infected mice showing cellular infiltration (arrows) and vacuolation (asterisk); and melatonin-treated groups; × 20 objective lens. **(B)** Brain cortex from PbA-infected mice showing infiltrated areas (arrows_×60 objective lens), dilated vessels (DV_×40 objective lens), and occluded vessels (OV_×60 objective lens). **(C)** Data quantification of dilated vessels, occluded vessels, and hemorrhagic foci/field. Images were analyzed in a double-blind manner; *n* = 10 animals/group (bars, 50 μm). *p < 0.01 vs control; **p < 0.05 vs control; ^#^*p* < 0.01 vs PbA; ^*##*^*p* < 0.05 vs PbA.

**Figure 6:**
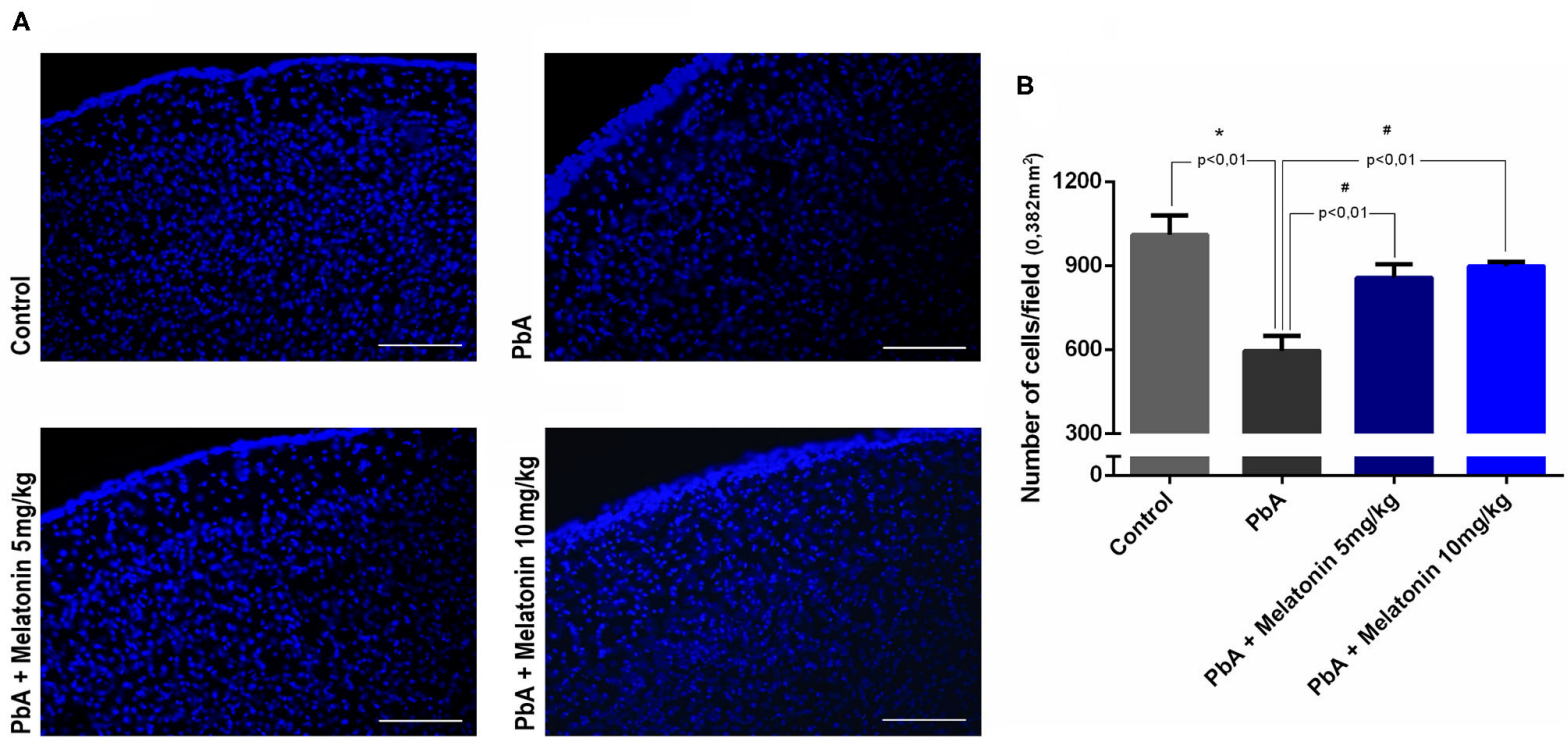
Analysis of cell number in brain sections using DAPI staining by fluorescence microscope (× 20 objective lens). **(A)** The most representative fields of brain cortical area are shown (scale bar, 50 μm). **(B)** Quantification of DAPI nuclear stain in distinct groups using ImageJ software. Data represent mean ± SD of three independent experiments; *n* = 10 animals/group. *p < 0.01 vs control; ^#^*p* < 0.01 vs PbA.

Histological analysis of brain cortical slices showed a disorganized parenchyma with evident acidophilic nuclear retraction and vacuolation at 6 days post-infection. Cellular infiltration, vascular dilatation, occluded capillaries, and hemorrhagic areas were also observed and quantified at this stage of the disease (Figures 5B,C). All these pathological changes were ameliorated by the treatment with both melatonin doses (Figures 5A,C). DAPI staining demonstrated that the number of cells was significantly reduced in the cortex of PbA-infected mice at 6 days post-infection. In contrast, the treatment with melatonin at 5 and 10 mg/kg protected the cortical tissue from this cellular loss (Figure 6A), maintaining the number of cells near the control group (Figure 6B). Thus, we demonstrated that melatonin treatment has prevented cell loss in the brain cortex of PbA-infected mice.

### Melatonin Treatment Attenuated Motor Behavioral Abnormalities Induced by PbA-Infected Mice

**Figure 7:**
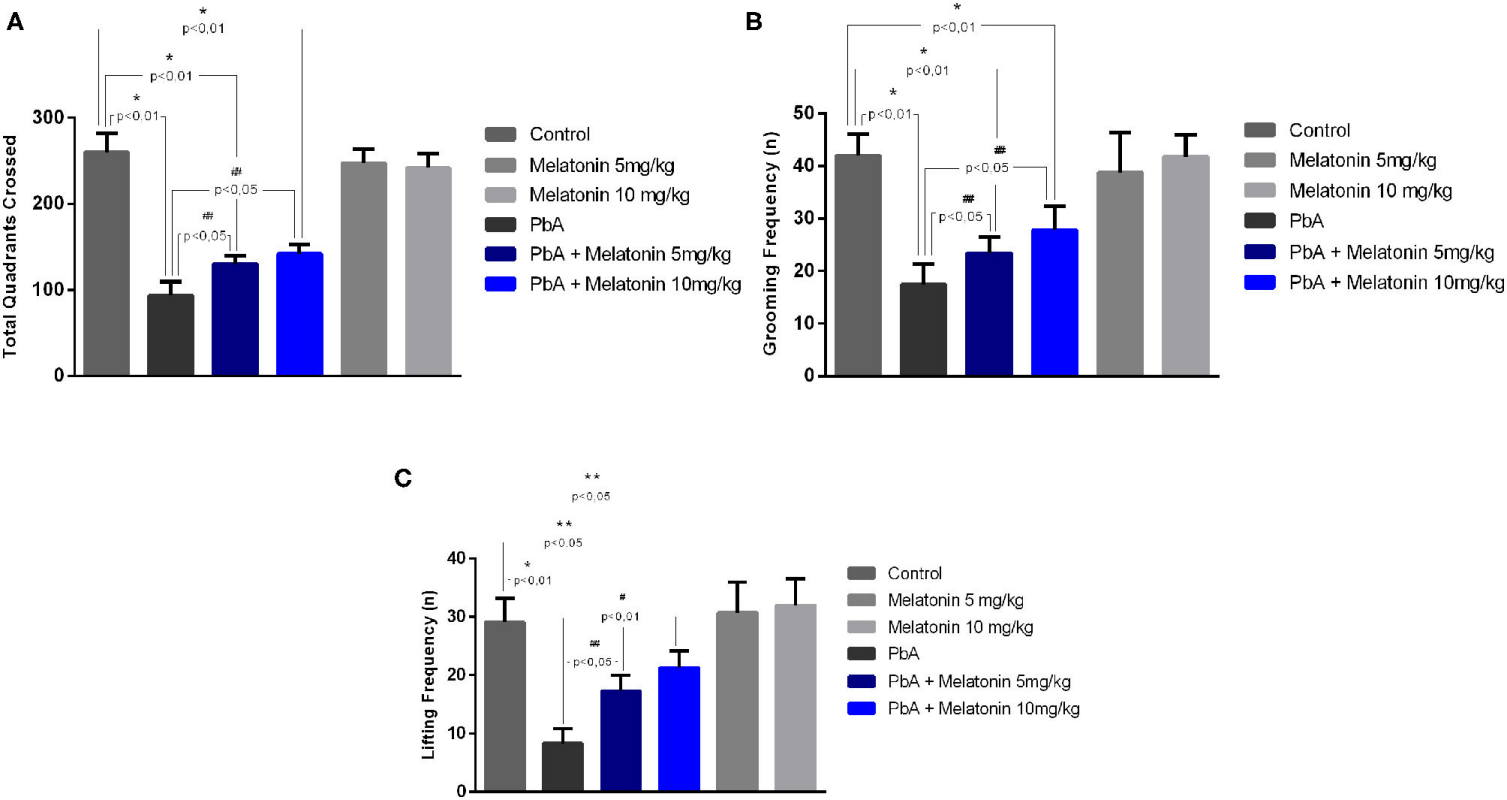
Protective effect of melatonin against motor impairment in PbA-infected mice at 6 days post-infection. Number of crossed quadrants **(A)**, grooming frequency **(B)**, and lifting frequency **(C)** were recorded and quantified. The experiment was repeated three times (*n* = 10 animals/group), and the values are given as the mean ± SD. *p < 0.01 vs control; **p < 0.05 vs control; ^#^*p* < 0.01 vs PbA; ^*##*^*p* < 0.05 vs PbA.

To evaluate the protective effect of melatonin on motor impairment, an open field test was performed in late stage of the disease on day 6 post-infection. On the proposed test, parameters such as lines crossed, rearing, and grooming behavior were measured in the distinct groups and illustrated in Figure 7. On day 6 post-infection, PbA-infected mice exhibited significant decreased locomotor activity and gait dysfunction by crossing a smaller number of squares as compared to uninfected mice, whereas melatonin-treated mice at different doses (5 and 10 mg/kg) showed a marked improvement in total locomotor activity by the increased number of crossed squares as compared to the PbA-infected mice (Figure 7A). Moreover, the uninfected group treated only with melatonin (5 and 10 mg/kg) showed no significant behavioral alteration compared with the control group, indicating that, in this condition, melatonin did not modulate the excitability of the CNS (Figure 7).

Additional measures of motor impairment such as the number of rearing and self-grooming were also analyzed. Both parameters were also found to be significantly decreased in PbA-infected mice as compared to the uninfected control group, and melatonin elicited significant improvement in these motor indicators (Figures 7B,C). Taken together, these behavioral results indicate that motor dysfunctions induced by ECM can be prevented by the treatment with melatonin.

## Discussion

In addition to being the main autocrine neurohormone released by pineal gland, melatonin has also been reported with anti-inflammatory and antioxidant effects (Tan et al., 2003; Esposito and Cuzzocrea, 2010; Acuña-Castroviejo et al., 2014). Moreover, this neurohormone has been shown to be neuroprotective against CNS injuries by its anti-apoptotic action (Mésenge et al., 1998; Ali and Kim, 2015; Shukla et al., 2019). CM is a devastating disease often responsible for high mortality rates. Clinical studies reveal that most survivors may not fully recover from persistent motor-neurocognitive impairment despite worthy recent advances in available antimalarial drugs (Murphy and Breman, 2001; Carter et al., 2005). Herein we report, for the first time, that melatonin treatment is able to ameliorate clinical signs and neurocognitive dysfunctions in PbA-infected mice, resulting in protection from ECM outcome. Murine experimental CM is characterized by BBB breakdown, brain edema, and parenchyma lesions, which lead to damage within the CNS (Bagot et al., 2002; Martins et al., 2009a). The outcome of CM is established during the acute phase of the PbA-infection when parasitemia reaches rates between 10 and 15%. At this stage, mice display characteristic clinical signs such as paralysis of the limbs, poor reflex, deviation of head, spontaneous rolling over, convulsions, and coma (Ampawong et al., 2014). In addition, while PbA-infected animals presented neurological symptoms and die between days 8 and 11 post-infection, melatonin-treated mice exhibited enhanced survival from acute infection, and most of them died between 15 and 21 days post-infection with no signs of neurological dysfunction. Our results suggested that melatonin suppresses CM development, and considering that animals showed elevated levels of parasitemia in the end stage of the disease (as described in Figure 1B), melatonin-treated animals probably died of severe anemia as previously described in anterior reports (Dende et al., 2015). All of these protective effects elicited by melatonin treatment were not associated with reduction of parasitemia levels and body weight, suggesting that melatonin has exerted its protective effect mainly on the CNS of infected mice.

The fact that melatonin did not alter the parasitemia at any stage of the disease could be explained by the fact that in *P. berghei* infection, the life cycle of the parasite is not influenced by the melatonin levels developing an asynchronous pattern of infection. Bagnaresi et al. (2009) demonstrated that the inhibition of melatonin receptor and the treatment with melatonin did not modify the parasite load in the livers of mice infected with *PbA* sporozoites.

There is a sturdy connection between the breakdown of the BBB and CM pathogenesis in both human and mouse experimental models (Lou et al., 2001; Medana and Turner, 2006; Dunst et al., 2017). Moreover, cerebral edema resulting from enhanced BBB breakdown is also a notorious feature of HCM and ECM (Thumwood et al., 1988; Penet et al., 2005). Although parasitemia levels were relatively high in melatonin-treated PbA-infected mice, there was a relevant reduction in BBB leakage and brain edema after melatonin treatment on day 6 post infection. Additionally, melatonin treatment also attenuates brain histological damages induced by PbA infection. Although our data demonstrated that the treatment with melatonin reduces the disarrangement in the brain cortex with typical and suggestive areas of leukocyte infiltration, additional studies using CD45 and CD8^+^ T cell staining need to be performed to better characterize this effect. This provides evidence that melatonin treatment could effectively reduce brain neuroinflammation associated with ECM pathology even in the presence of elevated parasitemia levels.

Furthermore, melatonin is an amphipathic molecule, which makes it suitable to cross not only BBB but also several other cellular compartments such as endoplasmic reticulum, mitochondria, and nucleus (Guha et al., 2007; Cardinali et al., 2013; Ding et al., 2015). The mechanisms through which melatonin protects BBB breakdown and subsequent brain edema during CM onset might be associated with the regulation of adhesion proteins in cerebrovascular endothelial cells, although additional studies need to be done to confirm our hypothesis.

We cannot exclude the fact that melatonin neuroprotection could be associated with its antioxidant effect once previous studies have already demonstrated that daily administration of melatonin restores antioxidant capacity and inhibit the production of pro-inflammatory cytokines in an experimental model of diabetic retinopathy (Negi et al., 2011). In addition, melatonin is able to decrease the expression of NADPH oxidase isoforms Nox2 and Nox4, reduces reactive oxygen species generation, and inhibits apoptotic cell death in a rat model of cerebral ischemia (Li et al., 2014).

In mouse models of newborn hypoxic–ischemic brain injury, melatonin exerts a neuroprotective role by the activation of M1 melatonin receptors, inhibition of mitochondrial cell death pathways, and astrocytic activation. Moreover, studies demonstrated that melatonin could potentially attenuate reactive gliosis and reduces microglial activation, which are remarkable and well-characterized events in CM pathogenesis (Cervantes et al., 2008; Sinha et al., 2017).

Besides, we also observed that melatonin treatment protected CM mice from long-term neurocognitive and motor impairment such as gait and motor dysfunction. The open field test is a useful method to measure poor gait and ambulation in rodent models of CM, and in the present study, PbA-infected mice that received melatonin performed as well as uninfected control mice. According to our behavioral analysis, melatonin significantly improved motor impairment in PbA-infected mice. A previous report has already described that chronic melatonin therapy attenuated D-galactose-induced memory and neuronal impairment by decreasing neurodegeneration through the activation of RAGE/NF-κβ and JNK signaling pathways (Ali et al., 2015).

Interestingly, a study also demonstrated that the deficiency of endogenous melatonin exacerbates the neuronal damage in traumatic brain injury patients, providing notable evidence of the exogenous administration efficacy. In agreement with our study, exogenous administration of melatonin by intraperitoneal injection decreases brain edema and infarct volume and ameliorates neurological deficits in mice brain injury in a single or repeated dose of 10 mg/kg (Reiter et al., 2003). Due to its functional diversity, exogenous melatonin has been investigated as a compatible candidate for the treatment of distinct disorders such as neuroinflammatory conditions and neurodegenerative diseases, but no studies have described the neuroprotective action of melatonin on the brain dysfunction induced by severe malaria (Samantaray et al., 2003; Singhal et al., 2012; Rosales-Corral et al., 2017; Su et al., 2017; Shukla et al., 2019).

In summary, our results in an experimental model of CM suggest that melatonin treatment may improve survival and BBB integrity and prevent neuromotor impairment in mice infected with PbA, without altering parasitemia levels. Moreover, the exact mechanism by which melatonin exerts its neuroprotective effect should be further investigated.

## Conclusions

In conclusion, the present study demonstrates for the first time the neuroprotective role of melatonin against BBB breakdown and behavior impairment evoked by CM. In this way, the current data represent an important further pre-clinical evidence that melatonin could be an efficient adjuvant for CM treatment.

## Data Availability Statement

The raw data supporting the conclusions of this article will be made available by the authors, without undue reservation, to any qualified researcher.

## Ethics Statement

The animal study was reviewed and approved by Animal Ethics Committee of the Federal University of Para (Protocol number: 6211241117/UFPA).

## Author Contributions

BA performed and acquired all the experimental data. NK performed the histological analyses. MT and LA performed and analyzed the behavioral data. NM performed the extravasation dye method. AP revised the manuscript and statistical analysis. SM contributed to the final drafting of the article. EB provided technical and material support. AH analyzed and interpreted the data. KO conceived, designed, and supervised the study.

## Conflict of Interest

The authors declare that the research was conducted in the absence of any commercial or financial relationships that could be construed as a potential conflict of interest.
